# Mechanical Behavior of Laminated Glass Polymer Interlayer Subjected to Environmental Effects

**DOI:** 10.3390/polym14235113

**Published:** 2022-11-24

**Authors:** Jonathan T. Knight, Alaa A. El-Sisi, Ahmed H. Elbelbisi, Michael Newberry, Hani A. Salim

**Affiliations:** 1 Civil and Environmental Engineering, University of Missouri, Columbia, MO 65211, USA; 2 Civil Engineering, Southern Illinois University Edwardsville, Edwardsville, IL 62026, USA; 3 On Leave from Structural Engineering, Zagazig University, Zagazig 44519, Egypt; 4 Blast Research Technical Lead at Battelle, Air Force Civil Engineer Center (AFCEC/CXAE), Tyndall AFB, Tyndall, FL 32403, USA

**Keywords:** environmental degradation, laminated glass interlayer, accelerated weathering

## Abstract

It is known that weathering action has a significant impact on polymer interlayer materials, and previous studies have evaluated certain aspects of weathering such as temperature, humidity, and UV radiation. In this paper, the environmental effect on the mechanical properties of the virgin and cured/processed polymer interlayer materials will be studied. Three polymer interlayer materials were focused, i.e., Polyvinyl butyral (PVB), Ethylene-vinyl acetate (EVA), and Ionomer (SG), due to their industrial interest. Testing setups were designed to apply the environmental effects and perform mechanical testing on the polymeric materials. Four environmental effects were studied, including water submersion (E1), constant high temperature (E2), cyclic temperature with low relative humidity (E3), cyclic temperature, and relative humidity (E4). After the exposure of these materials to these environmental effects, the samples were prepared and mechanically tested. Uniaxial tests were performed under static and high strain rates (around 45^−1^). It was found that under dynamic load, the properties of EVA such as the strength, maximum strain, and the toughness were not significantly affected by the environmental effects. SG5000 properties were significantly affected.

## 1. Introduction

Weathering action has a significant impact on the mechanical properties of polymer interlayers and can modify their behavior as a consequence of temperature, humidity, and solar radiation. Currently, there are three main interlayer materials that are widely used in the laminated glass industry. Polyvinyl butyral (PVB), Ethylene-vinyl acetate (EVA) and ionomer (SG). Polyvinyl butyral (PVB) is prepared from polyvinyl alcohol by reaction with butyraldehyde. Ethylene-vinyl acetate (EVA) is the copolymer of ethylene and vinyl acetate, while ionomer (SG) is a polymer composed of repeat units of both electrically neutral repeating units and ionized units covalently bonded to the polymer backbone as pendant group moieties.

Many studies demonstrate the significant influence of weathering on the physical and mechanical properties of Polyvinyl butyral (PVB), the most commonly used interlayer in laminated glass. For example, Saad et al. investigated the behavior of PVB following UV radiation and concluded that UV has a significant impact, showing that during UV irradiation of PVB, cross-linking predominates [1]. 

Andreozzi et al. investigated the effects of humidity, thermal cycles, and UV radiation on PVB, concluding that temperature had little to no impact; humidity impacted adhesion more than the bulk response, and UV had the most significant impact on the mechanical behavior [2]. The UV caused a dramatic stiffening of the PVB. While stiffening might be considered beneficial in some cases, it can cause the PVB to exhibit brittle behavior, which is counter-productive for energy absorption. Stiffening also reduces adhesion, which is necessary for the interlayer to contain glass shards after the window break. In all three cases, following specimen conditioning, Andreozzi et al. measured rheological properties using an oscillatory test [2]. Tensile tests, which are used in the study outlined in this paper, could produce new and possibly different conclusions than oscillatory tests. 

Some authors studied the mechanisms of degradation of Ethylene-vinyl acetate (EVA) and ionomer (SG) as well. Serafinavicius et al. subjected glass beams laminated with SG, PVB, and EVA to a combination of humidity, high temperature, and UV radiation [3]. All tests were carried out in a climatic chamber. The temperature levels of +200 °C, +300 °C, and +400 °C were controlled automatically at 24 h for each temperature level, with 72 h in total loading time. Humidity inside the chamber was controlled at 50%. Long-term four-point bending tests were carried out. The results were compared in a load path diagram. It was found that temperature aging effects have the greatest impact on all laminated glass specimens and the humidity aging effect has a minimal impact. The aging effect of UV radiation causes a slight hardening of the interlayer, and some deflection difference appears at a higher temperature of +400 °C. The combined effect of temperature, humidity, and UV has a similar impact as UV radiation [3].

Delincé et al. investigated the effect of artificial weathering, particularly humidity and UV radiation, on shear-bond properties of PVB and SentryGlas^®^ Plus (SGP) using different types of mechanical tests [4]. These experiments aimed to compare local effects from shear tests to global effects from bending tests. Laminated glass plates measuring 300 × 300 mm were subjected to artificial weathering prior to drilling the cylindrical samples to be used in shear tests. Laminated glass plates of 1100 × 360 mm for bending tests were subjected to similar weathering exposures. For both types of artificial weathering, UV and humidity, evaluation of samples was made not less than 24 h after the end of the weathering process, according to the ISO 12543-4. A visual evaluation of signs of delamination was done in all cases, and a measurement of the light transmittance before and after exposure to UV radiation was done only for 300 × 300 mm samples. No defect according to the evaluation criteria of the standard was noticed for the tested samples. The main conclusion is that mechanical tests can be relevant to measure the effects of weathering on shear-bond properties, complementary to visual evaluation prescribed in standards, to calibrate design values on the basis of statistical analysis. 

Butchart and Overend described the results of an experimental campaign of peeling tests. The peel tests were performed to investigate adhesion under different moisture levels [5]. Peel tests were performed on specimens of PVB laminated between a layer of glass and a layer of foil backing. The investigations show that in the presence of water, the adhesion between the glass and interlayer was less than half that observed in dry conditions. 

Weller and Kothe carried out different aging scenarios on modified PVB, thermoplastic polyurethane (TPU), ionomer (SG), and EVA to assess the long-term stability, such as a temperature storage test, a climatic stress test, and a test under aggressive media and high irradiation [6]. These aging tests with small-scale test specimens affected both the appearance and the material properties. It was concluded that the best performance interlayer materials after the different aging tests are SG and TPU. These materials are best suited for long-term use as a laminated glass interlayer for both indoor and outdoor applications. 

Ensslen reported an extensive analysis of the behavior of laminated glass specimens subjected to weathering action: some specimens were subjected to UV radiation in a solarium, some underwent temperature and humidity degradation cycles, and some were simply exposed to outdoor weathering for two years, in different climates [7]. The comparison between the specimens artificially weathered and the ones exposed to the outdoor weathering was made via monotonic shear tests. Experimental investigations showed that moisture penetration of the PVB interlayer on the glass edges has a negative impact on the durability of laminated glass. This results in an impairment of the shear stiffness as well as the bond strength. The aging of the interlayer due to UV radiation and high air temperatures, depending on its duration and intensity, leads to stiffening of the material properties, but not to impairment of structural safety [7].

Antolinc et al. performed a three-point bending test on laminated glass at elevated temperature in an environmental chamber [8]. The tested specimens were made of two fully tempered glass plates bonded with EVA and PVB interlayers. The tests were conducted at 23 °C, 35 °C, and 60 °C after the specimens had reached the defined contact temperature. It was found that laminated glass with the EVA interlayer exhibits more favorable overall behavior at the elevated temperature in comparison to the specimen with the PVB interlayer. The only deficiency of the EVA interlayer is that it began to tear at the temperature of 60 °C. Further research on the bending of laminated glass with smaller temperature steps is recommended, as well as at temperatures below room and sub-zero temperatures [8].

Martín et al. conducted high strain rate tests on seven different polymer interlayers, including three different PVB products, one SG product, two EVA products, and a TPU product at three different strain rates [9]. The mechanical and optical properties of unaged specimens are compared with specimens exposed to thermal cycles, high temperatures, and moisture. The unaged specimens of PVB and SG had the highest stiffness, EVA had the highest ductility, and PVB and SG had the highest tensile strength. In addition, EVA and TPU were less affected by aging factors and strain rate [9].

Sahmani and Fattahi performed modeling and analysis for size-dependent buckling and post buckling behavior of cylindrical nano panels made of silicon under axial compression in thermal environments. It was found that the thermal environments have no influence on the value of the minimum load of the post buckling domain and associated maximum defection of an axially loaded nano panel [10].

Several works focused on the mechanical properties of the interlayer material under static and dynamic strain rates [11,12,13,14,15,16,17,18,19,20,21,22,23,24]. The most common polymer material used in safety LG is PVB. PVB’s mechanical response is highly time-dependent, and it can elongate to several times its initial length. There was a wide range of strain rates at which PVB was tested in previous works [25,26]. However, strain rates observed in blast testing were in the range of 1/s to 10/s [26]. Moreover, the tearing of the 0.75-mm (0.03-inch) thick interlayer was observed at a 13% strain at a strain rate of 40 s^−1^ [26]. A strain rate of 40 s^−1^ was suggested for typical blast-resistant laminated glass for PVB material at 23 °C [25]. Finite element analysis was performed by Nawar et al. to investigate the strain rates at failure for different window systems which were tested in the field using live explosives. It was found that the strain rate was 40 s^−1^ at failure for the windows that experienced tearing and was less than 20 s^−1^ for the windows that did not fail [27]. 

In addition, the environmental effects such as exposure to temperature cycles, moisture, air, and light, can significantly affect its mechanical characteristics of these materials. Though previous research has evaluated the quasi-static performance of interlayer materials subjected to environmental effects, there is a lack of evaluation of the high-strain rate performance of environmentally affected interlayer materials. Furthermore, the effects of the curing process on the response of interlayer materials have not been studied. In this research, the quasi-static and dynamic responses of various virgin and cured interlayer materials subjected to environmental effects are presented. The results of this research are expected to enhance understanding of the effect of the environmental conditions on the long-term design of the LG windows against static and dynamic loads. The samples were subjected to environmental effects such as water immersion, constant and cyclic temperature, cyclic and humidity. PVB, EVA, and SG were evaluated under high strain rates and quasi-static loading with and without environmental effects.

## 2. Experiments

In this section, the experimental program will be explained. Details such as the materials under investigation, the testing equipment and testing procedure will be described.

### 2.1. Polymeric Interlayer Materials

Several interlayer materials are available on the market, and these are commonly used by glass processors to produce their laminated glass systems. These materials are usually delivered to the manufacturers in rolls of the sheet with specific thickness. Table 1 lists the properties of the polymer sheets used in this study. The cured/processed materials tested were cut from 1.5-mm thick (0.06 in) PVB and EVA sheets, and 0.89-mm (0.035 in) thick SG5000 sheets. The virgin materials tested were cut from 0.76-mm thick (0.03 in) PVB and EVA sheets, and 0.89-mm (0.035 in) thick SG5000 sheets.

There are fabrication processes for interlayer material that could cause a change in the material properties from the virgin state received from the manufacturer. In most cases, this is due to the lamination procedure that is used to adhere the product to the glass system. These effects will be tested by performing the lamination procedure on the test specimens. Lamination will be accomplished by following the manufacturer’s process which can vary for the different materials/manufacturers. 

### 2.2. Testing Matrix

A group of dynamic and static tests will be performed on laminated glass interlayer materials subjected to environmental effects. The environmental effects include the water absorption (E1), the constant high temperature (E2), the impact of cyclic temperature (E3), and the cyclic temperature and humidity (E4). The testing will be performed in two stages. In the first stage, the material will be subjected to the environmental effects shown in Table 2.

In the second stage, the environmentally affected samples were tested to evaluate their mechanical properties, see Table 3. All the tests in this study are uniaxial tension tests under static and high strain loads.

## 3. Environmental Effects

The environmental study in this paper includes accelerated weathering effects such as high temperature and water immersions. In this section, the procedures used to apply these effects on the samples are detailed.

### 3.1. Water Immersion

**Figure 1:**
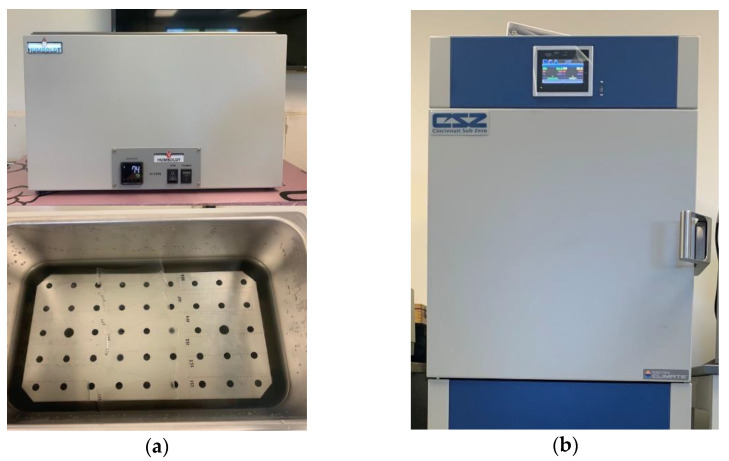
Accelerated Weathering Equipment: (**a**) Water immersion bath and (**b**) CSZ environmental chamber.

The first effect is water immersion; the water absorption for each material can be determined following the standards ASTM D570-98 and ISO 62 [28,29]. To perform quasi-static and dynamic tests on the material after immersion, the 60 × 60 mm test specimen specified in the standards is not acceptable. Therefore, 5 × 1.5 inch and 9 × 2-inch rectangular specimens are used for quasi-static and dynamic testing, respectively. The specimens are weighed on a scale to the nearest 0.1 mg then placed in a thermostatic water bath, Figure 1a. The water bath is filled with distilled water at a temperature of 73.4 ± 1.8 °F until the water level is approximately one inch above the specimens. The specimens are removed and weighed to the nearest 0.1 mg at intervals of 1, 2, 4, 8, 16, 24, 48, 96, and 168 h following the procedure used by [12]. After 168 h, the specimens are dried in an oven at 40 °C for 24 h and then re-weighed to verify total moisture content. 

### 3.2. Environmental Chamber Effects

Three different environmental effects are done by using Cincinnati Sub-Zero MCHS-3 environmental chamber, namely, E2, E3, and E4. In the E2 environmental effect, an isothermal temperature program at 60 C based on UNI EN ISO 12543–4 standard was used for 16 h [28]. The relative humidity was kept constant at 10% during this test.

On the other hand, in E3, a dynamic temperature program is established for thermal cycling tests, varying the temperature between 30 °C and 50 °C at a constant cooling and heating rate of 3.0 C/min. The relative humidity was kept constant at 10% during this test. A total of 80 cycles were performed following this methodology. In E4, temperature and humidity cyclic programs were established. The program is similar to the E3 program except that the humidity was not kept constant. The relative humidity varied with increasing and decreasing temperatures. Temperature and humidity varied between 30 °C at 10% relative humidity and 50 °C at 95% relative humidity. After applying the environmental effect, the specimens are kept in a desiccator while awaiting testing to keep out moisture. The standard testing coupons are cut from the rectangular specimens and tested following the standard procedures.

## 4. Mechanical Testing 

This section covers the next stage of the experimental study, which is mechanical testing.

The objective of this process is to evaluate the mechanical properties of the polymer interlay with and without the environmental effects. Sample preparation, testing equipment, and data acquisition system will be presented in this section.

### Specimen Preparation

**Figure 2:**
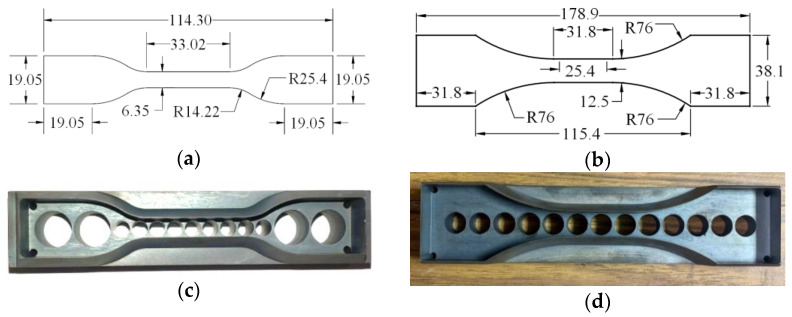

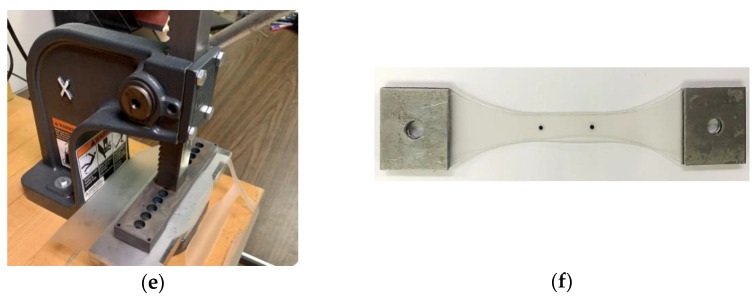
Specimen Preparation: (**a**) Static Specimen Geometry, (**b**) Dynamic Specimen Geometry, (**c**) Static Cutting Die, (**d**) Dynamic Cutting Die, (**e**) Specimen Stamping, and (**f**) Sample Marking. (Dimensions mm).

In this study, the static testing sample geometry was a standard Type IV specimen, as shown in Figure 2a, according to ASTM D638-10 [30]. However, modified specimen geometry was used for dynamic tests [31]. It was designed by modifying the Type I standard specimen (ASTM D638−10 2010), which is shown in Figure 2b. The modified geometry helped to increase the bonding area between the aluminum tabs and the interlayer polymer specimens to prevent the tearing of the specimens at the ends of aluminum tabs. To ensure the accuracy of the specimen dimensions, steel cutting dies were manufactured (see Figure 2c–e). Holes through the back of the cutting dies allowed for retrieval of the stamped specimens without crimping or tearing of the material. The 1-inch central gauge length, Lg, was marked with thin black lines/points using a permanent marker pen before testing to enable the strain to be monitored during the test using a high-speed camera, Figure 2f. Digital calipers were used to measure the thickness and width of the test section at three locations to an accuracy of 0.0005”.

A temperature control chamber was used to adapt the test temperature to ensure the isothermal condition during the mechanical testing process. The system contains an insulated enclosure (chamber) that was made from thick foam and designed to surround the sample to provide isothermal conditions. The chamber has an inlet and outlet, where the air inside the chamber is circulated through a heat exchanging unit. The heat exchanging unit includes insulated ducts, a fan, and a cooling coil. A liquid at a low temperature is circulated inside the coil by using a chiller. 

## 5. Static Testing Setup

For the quasi-static tensile test, an electromechanical static testing frame was used. The total travel distance for this apparatus is only 18 inches. This device has accurate load cells and a Linear Variable Differential Transformer (LVDT) to measure the total extension of the specimen. The data acquisition system is also attached to the apparatus, which transfers the test data to a specialized LabView 2020 program. In addition, the total distance between the two grips is also recorded. The deformation of the gage length of the specimen was calculated by using a high-resolution camera.

### 5.1. Drop Weight Testing Equipment

**Figure 3:**
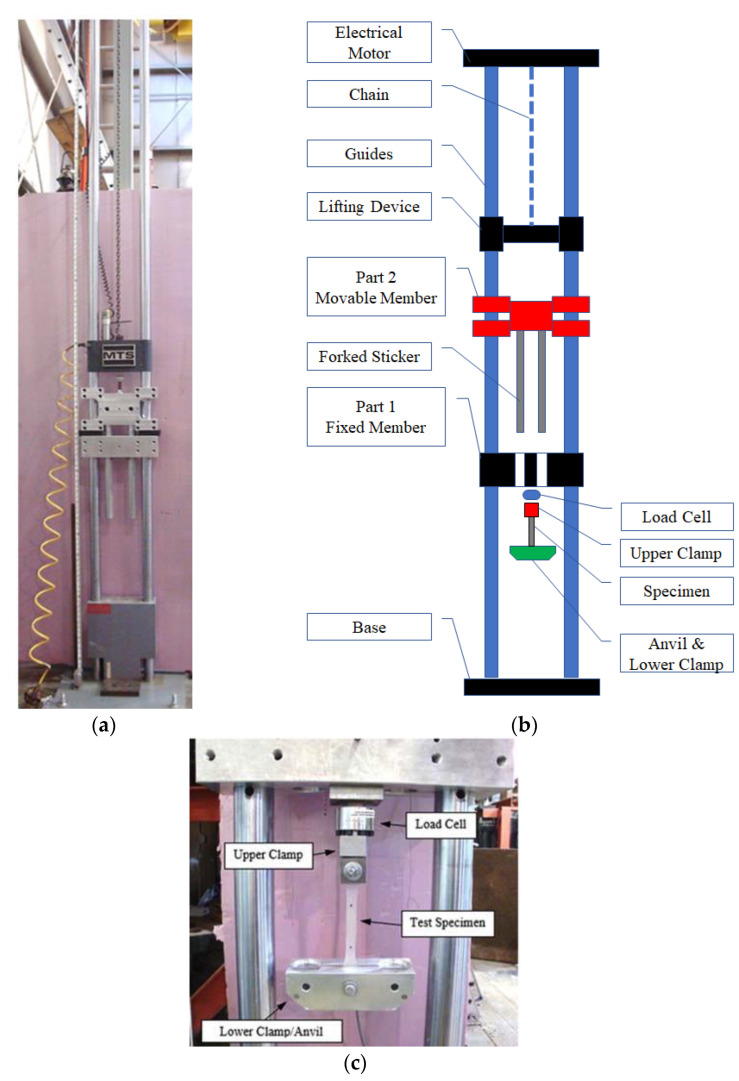
Drop Weight Test Set-Up: (**a**) Drop Weight Testing Frame, (**b**) Drop Weight Frame Schematic Diagram, and (**c**) Sample Mounting.

The high-strain rate tensile testing in this research was performed using a drop-weight apparatus, see Figure 3a,b. The apparatus consists of a forked striker and an anvil that create an impact load to produce an extension to a tensile specimen at an acceleration above zero, Figure 3c. A piezoelectric load cell (Omegadyne model LC213-500) was used to measure the load and calculate the engineering stress at a rate of 3000 data points per second. The elongation of the specimens was monitored during the test using a high-speed camera (model SC1 manufactured by edgertronic) at a recording rate of 3000 frames per second. The load from the piezoelectric load-cell was recorded by a National Instruments USB-6351 data acquisition system.

### 5.2. Data Processing

Images of the sample deformation captured by a high-speed camera were post-processed to calculate the strain in the sample. A software was developed in-house, which was used to track the position of the gauge length lines during the loading event. More details about this software are found the following references [32,33]. The engineering strain was then calculated using the original length, determined from the frame just before the start of loading, and the difference in length in all subsequent frames. 

**Figure 4:**
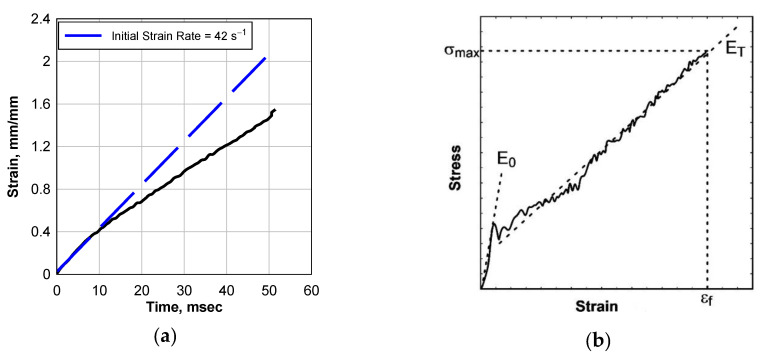
Data Analysis: (**a**) Typical Engineering Strain–Time Response, and (**b**) Typical Stress–Strain Curve.

A typical engineering strain–time response for a PVB drop weight test is shown in Figure 4a. The black curve shows the experimental data, and the blue curve is a linear curve fit. The strain of the sample was calculated from tracking markers on the sample in images captured by the high-speed camera. In general, the strain–time response is linear; however, slight softening occurs happens sometimes. Therefore, the experimental strain rate, ${\dot{\varepsilon }}_{E}$, was calculated as the average slope of the strain–time curve. 

A typical engineering stress–strain response for a PVB drop weight test is shown in Figure 4b. The engineering stress was calculated by dividing the load by the original cross-sectional area of the gauge length of the specimen. Because the load was captured at the same rate as the images used to calculate strain, it was not necessary to interpolate the strain data to match the times of the load data. The maximum stress, σ_max_, and strain to failure, ε_f_, were calculated at the time of maximum force. Two moduli were also calculated: Young’s modulus, E_0_, which is the slope of the initial linear response, and the tangent modulus, E_T_., which is the slope of the elastic portion of the response. The strain energy, U, was calculated as the area under the stress–strain curve.

## 6. Experimental Results Interlayer Tests

In this section, the results of the experimental tests will be discussed, including the static and dynamic stress–strain relation, and the dynamic strain effect on the virgin and cured polymer interlayer materials; Table 4 lists all the sample. The stress–strain curves will be described by four terms, i.e., Term1- Term2- Term3- Term4.

- Term1 describes the material state, and it can be V for the virgin interlayer or C for cured/processed interlayer;
- Term2 describes the interlayer type, and it can be P for PVB, E for EVA, and S for SG5000;
- Term3 will describe the test type, and it can be S for Quasi-Static tests and D for dynamic tests;
- Term4 will describe the type of the environmental effect; it can be E0 for the control group or E1, E2, E3, and E4.

### 6.1. Control Group (E0)

All the materials under investigation were tested at room temperature (RT) as the control group for the other environmentally affected materials. Static tests at 2 in/s speed and high strain test at average strain around 45 s^−1^ were performed for these materials. In this section, the results of these tests will be investigated. 

**Figure 5:**
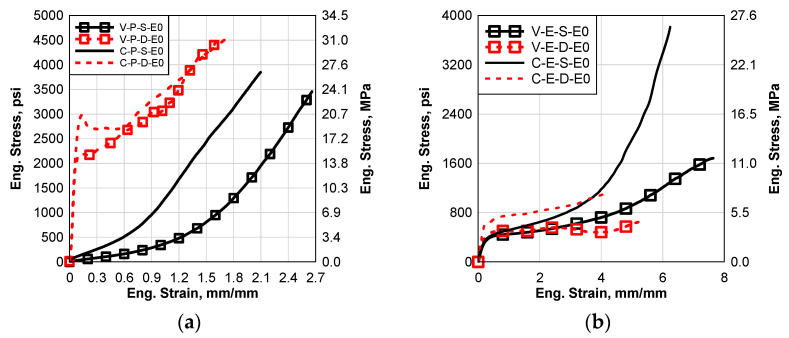

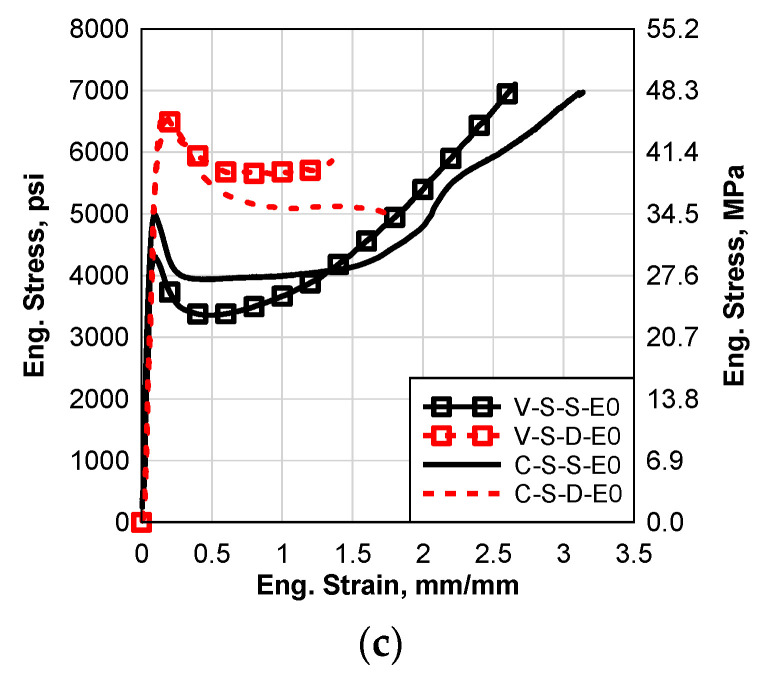
Static and Dynamic Stress–Strain Results of the Control Group (E0) for Virgin and Cured Materials: (**a**) Saflex Standard Clear PVB, (**b**) EVA EVGARD and (**c**) Kuraray SG5000.

Figure 5a–c show the load stress–strain relation of the three materials in the virgin and cured/processed states without any environmental effects. It can be seen that the static behavior of EVA and SG5000 showed a bilinear stress–strain relationship, Figure 5b,c. However, the PVB exhibited a hyperplastic performance, Figure 5a. EVA has the lowest strength among all materials while SG5000 has the maximum, Figure 5b,c. The high strain rate performances of the three virgin materials have a bilinear relation, Figure 5. The failure strains under the high strain rate are lower than the static case for all the virgin materials. The resistance of PVB and SG5000 increased significantly under the effect of the high strain rate, Figure 5a,c. However, the strength of virgin EVA was reduced significantly due to the effect of the high strain, Figure 5b. For SG5000, there is a sudden softening behavior that happened after the yield stress for both the static and dynamic tests, Figure 5c. The softening could happen due to the surface cracking of the SG material as SG material shows a higher brittle behavior compared to the other materials. This softening behavior causes the stress to drop significantly just after the initial yield strength. By increasing the strain, the stress returns to increase again until the failure. It can be concluded that all the used virgin materials are sensitive to high strain rates. Comparing the cured materials to the virgin materials, it can be observed that the curing process caused a hardening for the three materials in both static and dynamic cases, except the dynamic curve of SG5000. Comparing the cured curves to the virgin curves, it can be seen that the curing process caused a reduction in both failure strain for PVB and EVA, while it caused an increase in these values for the SG. Finally, the curing effect is much more significant in the static behavior than the dynamic behavior.

### 6.2. Humidity—Water Immersion (E1)

**Figure 6:**
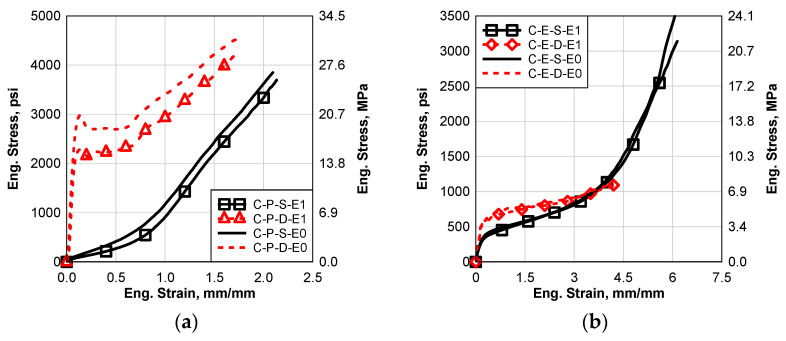

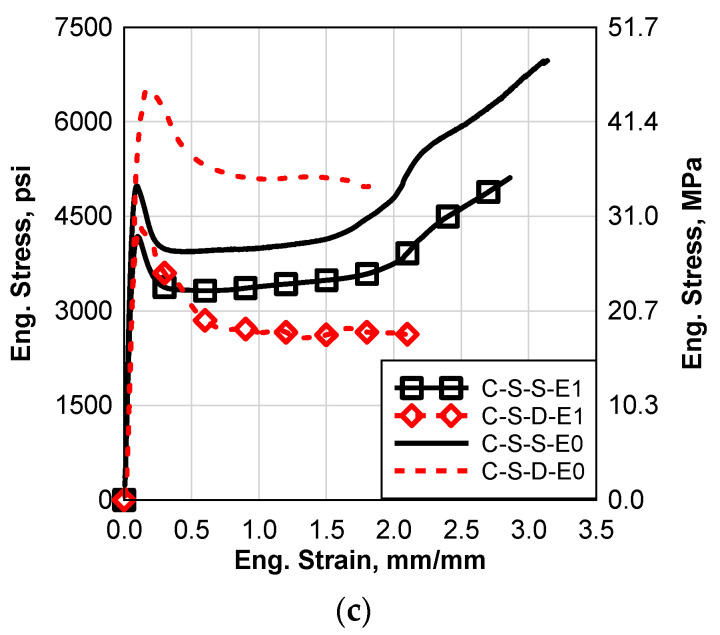
Comparison between Static and Dynamic Stress–Strain Results of the Groups E1 and E0: (**a**) Saflex Standard Clear PVB, (**b**) EVA EVGARD and (**c**) Kuraray SG5000.

The water immersion environmental effect (E1) was performed by immersing the samples in distilled water for about one week. The amount of water absorption was recorded over the week for all the materials. After that, the samples were dried in the oven and tested at room temperature. In the previous section, a comparison between the virgin and cured/processed material stress–strain behaviors was established Figure 6. In this section, the results of the cured/processed materials with and without the water immersion environmental effect will be presented and discussed. 

Static and high-strain tests were performed for all the materials. Figure 6 shows comparisons between the static and dynamic results of all the materials, with and without the environmental effects. E1 environmental effect caused degradation for all the materials. This degradation is more significant in the case of PVB and SG5000, as shown in Figure 6a,c. However, the EVA was slightly affected by the water immersion effect, Figure 6b. For PVB and SG5000, the water immersion environmental effect is more significant in the case of dynamic behavior than the static behavior, Figure 6a,c. For the SG5000, E1 affected the failure strain significantly, however, it only slightly affected the PVB and SG5000 strains. It can be concluded that the water immersion environmental effect caused a softening of all the materials, and this softening is larger in the case of SG5000 and lower in the case of EVA.

**Figure 7:**
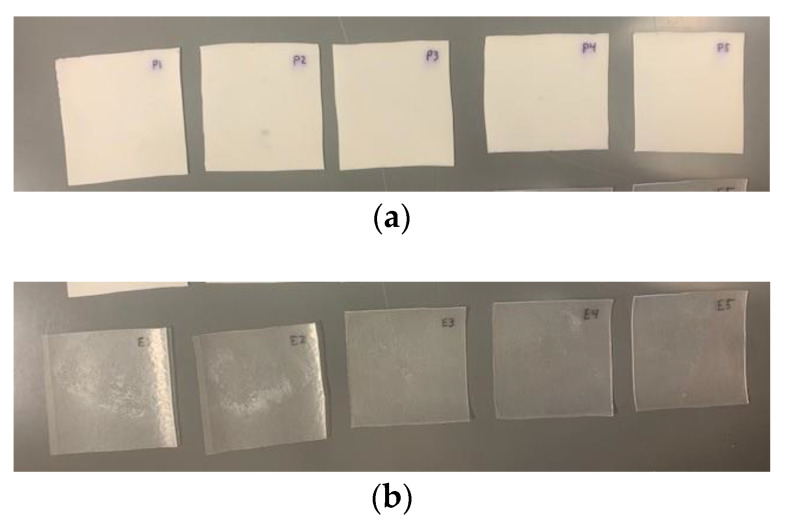
Immersed PVB versus EVA Visual Comparison: (**a**) Immersed PVB and (**b**) Immersed EVA.

For the physical properties, the PVB specimens became opaquer in the water, turning a milky white color instead of their typical clear, Figure 7. This opaqueness disappeared after drying the specimen in the oven at the end of the 168-h weighing period. This change could cause visibility issues for LG windows bonded with PVB due to humidity migration.

**Figure 8:**
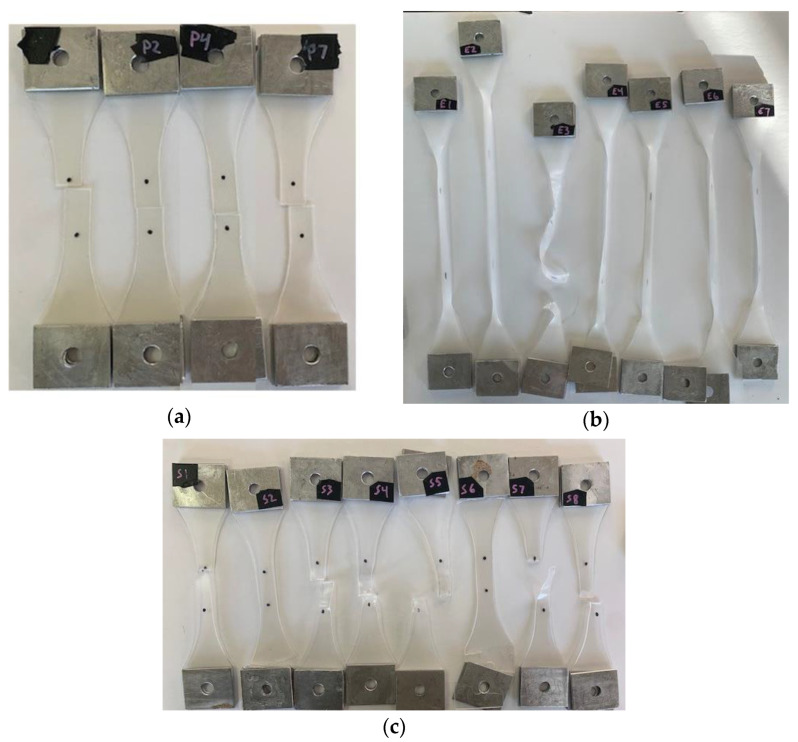
Water-Immersed Dynamic Specimens After Testing: (**a**) PVB, (**b**) EVA, and (**c**) SG.

The failure modes of the three materials were different as seen in Figure 8. The deformation of the PVB looked uniformly distributed over the sample until it was cut at the middles of the gage length. For EVA, the sample stretched much more than PVB and SG5000, then the straight area of the specimen turned white due to the excessive strain, and finally, it was broken. For SG5000, after the first peak or the yielding, A sudden reduction happened in the stress accompanied by a strain localization at the middle of the sample. The strain localized at the area until the end of the test. This reduction after the yield stress might be due to the surface cracking as the surface of the SG5000 looks brittle unlike the EVA and PVB.

**Figure 9:**
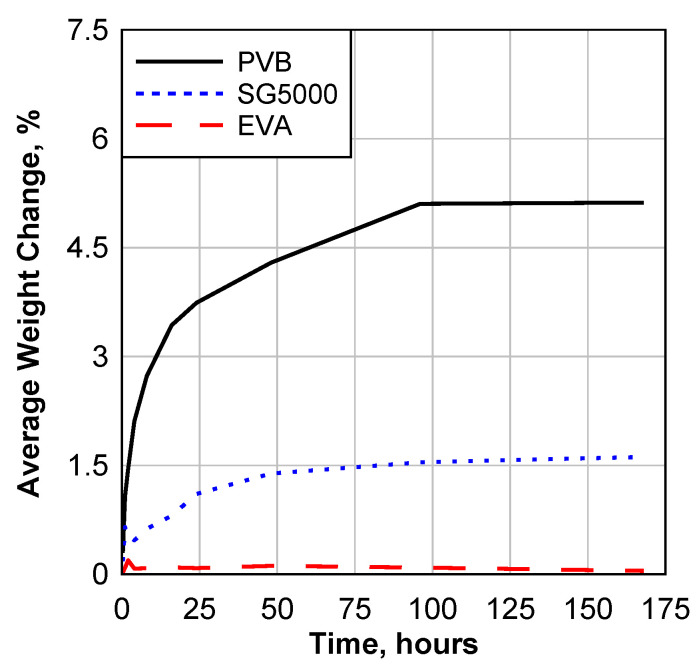
E1 Water Immersion of Processed Materials Data.

Figure 9 shows the average weight change due to the water immersion during the week. It can be seen that PVB has the maximum absorption followed by SG5000. EVA did not absorb much water, which explains why its performance was not significantly affected by this environmental effect. The maximum weight gain percentages were 4.9, 1.6, and 0.05% for PVB, SG5000, and EVA, respectively.

### 6.3. Constant Temperature—60 °C (E2)

**Figure 10:**
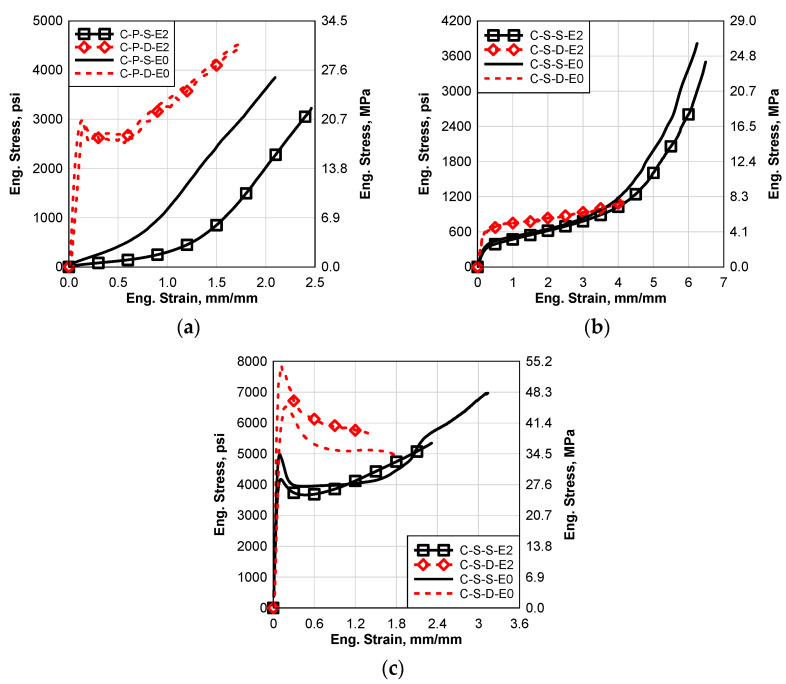
Comparison between Static and Dynamic Stress–Strain Results of the Groups E2 and E0: (**a**) Saflex Standard Clear PVB, (**b**) EVA EVGARD and (**c**) Kuraray SG5000.

In this section, the results of the processed materials with and without the high-temperature environmental effect will be presented and discussed. Static and high strain tests were performed for all the materials. Figure 10a–c show comparisons between the static and dynamic results of all the cured materials with and without the high-temperature environmental effects. It can be seen that the E2 environmental effect caused a softening for all the materials except the dynamic SG5000 results, Figure 10c. This change in material behavior is more significant in the case of PVB and SG5000 as shown in Figure 10a,c. However, the EVA was slightly affected by E2 as shown in Figure 10b. For PVB and EVA, the high-temperature environmental effect is more significant in the case of static behavior than dynamic behavior. However, it affected the dynamic behavior of SG5000 more than the static behavior. For the PVB static and SG5000 static and dynamic, E2 affected the failure strain significantly, however, it only slightly affected the EVA.

### 6.4. Cycle Temperature with Constant Low Humidity (E3)

**Figure 11:**
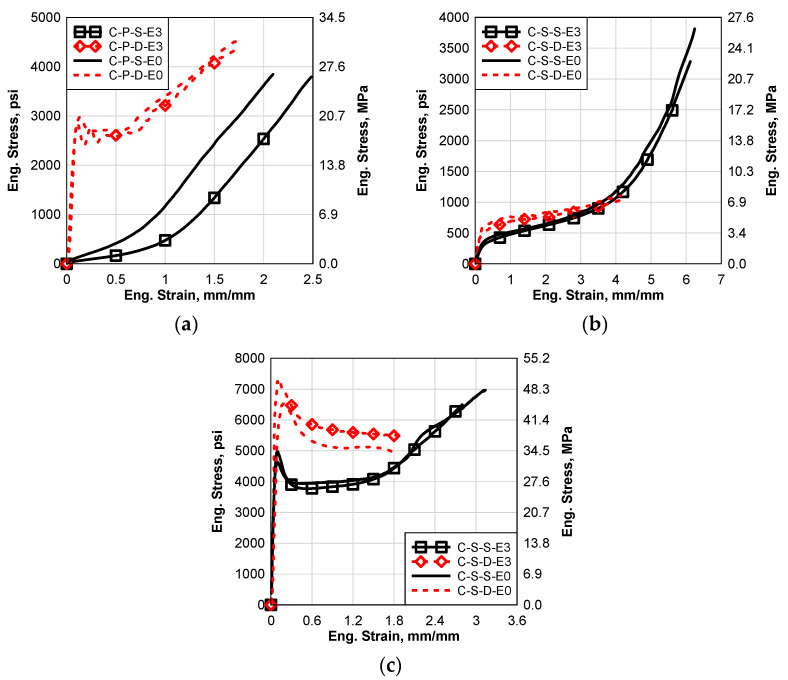
Comparison between Static and Dynamic Stress–Strain Results of the Groups E3 and E0: (**a**) Saflex Standard Clear PVB, (**b**) EVA EVGARD and (**c**) Kuraray SG5000.

The cycle temperature environmental effect (E3) was performed by using an accelerated weathering chamber. In this section, the results of the processed materials, with and without the cycle temperature environmental effect, will be presented and discussed. Static and high strain tests were performed for all the materials. Figure 11 shows comparisons between the static and dynamic results of all the materials, with and without the cycle temperature environmental effects, like E2; it can be seen that the E3 environmental effect caused softening degradation for all the materials except the dynamic SG5000 results; it caused hardening, as seen in Figure 11c. As well as E1 and E2, the change in material behavior is more significant in the case of PVB and SG5000, as shown in Figure 11a and c. However, the EVA interlayer was slightly affected by E3 as shown in Figure 11b. For PVB, the high-temperature environmental effect is more significant in the case of static behavior than dynamic behavior. However, it affected the dynamic behavior of SG5000 more than the static behavior. For the PVB static test and SG5000 static and dynamic tests, E3 affected the failure strain significantly although it only slightly affected the EVA.

### 6.5. Cyclic Temperature and Humidity (E4)

**Figure 12:**
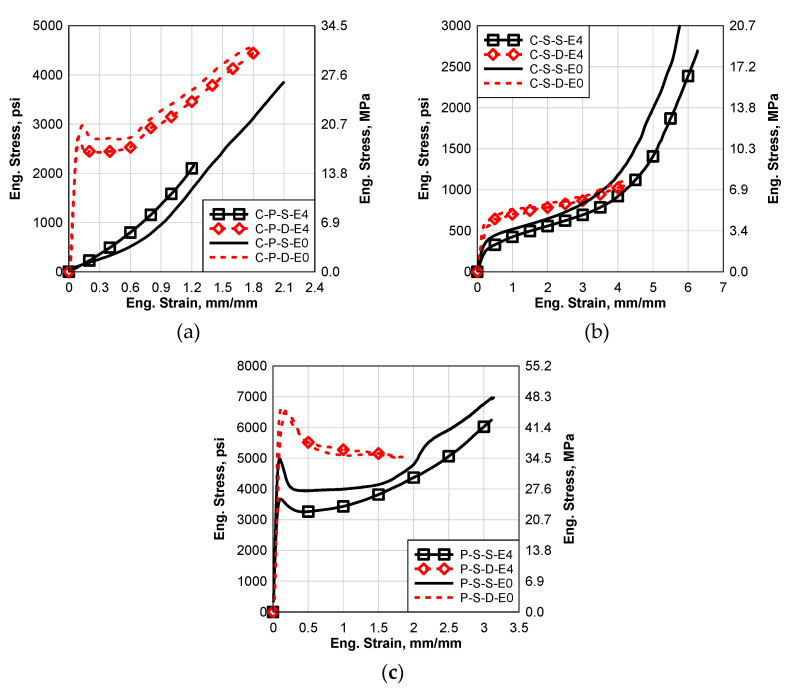
Comparison between Static and Dynamic Stress–Strain Results of the Groups E4 and E0: (**a**) Saflex Standard Clear PVB, (**b**) EVA EVGARD and (**c**) Kuraray SG5000.

In this section, the results of the processed materials, with and without the cycle temperature and humidity environmental effect, will be presented and discussed. Figure 12 shows comparisons between the static and dynamic results of all the materials with and without the E4 effects. It can be seen that the environmental effect of E4 did not significantly affect the dynamic stress–strain curves of SG5000 and EVA, Figure 12b,c; however, some effect was observed in the dynamic PVB curve, Figure 12a. The static performance was significantly affected for all the materials more than the dynamic performance. The static failure strain of the PVB was significantly reduced, although there is no significant effect in the other cases on the failure strain.

### 6.6. Comparison between Different Environmental Effects

**Figure 13:**
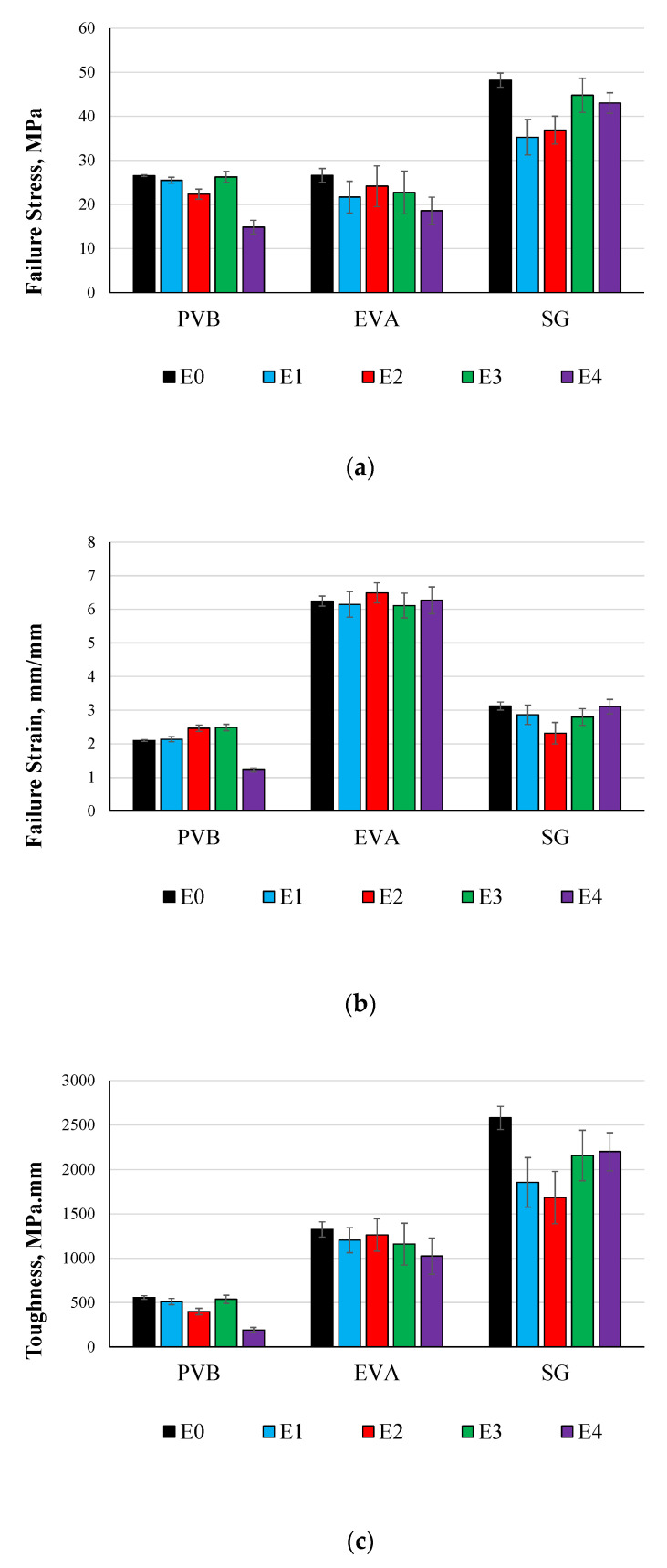
Comparison between Static Testing Results of Accelerated Weathering for Cured Materials: (**a**) Static Testing Failure Stress, (**b**) Static Testing Failure Strain and (**c**) Static Testing Toughness.

The previous sections focused on the overall comparison of the stress–strain relationship. In this section, the failure stress and strain, and the toughness will be compared and discussed for both static and dynamic tests. The toughness was considered for the area under the stress–strain curve up to the failure. Figure 13a shows a comparison between the maximum failure stress of all the materials under different environmental effects from the static tests. For all the cured materials, it can be seen that the four environmental effects caused a loss in strength with different percentages. For both PVB and EVA, the E4, which includes cycles of temperature and humidity, was the most effective environmental effect. E1 and E3 have nonsignificant effects on the strength of PVB, while E2 has the least effect on EVA. For SG5000, the material strength was significantly affected by E1 and E2, and slightly affected by E3. Figure 13b shows the maximum failure strains, where it can be seen that all the environmental effects caused an increase in the failure strain of PVB except E4, which caused a significant reduction in the PVB failure strain. All the environmental effects caused a reduction in EVA and PVB failure strains. The maximum reductions in EVA and SG were due to E4 and E1, respectively.

**Figure 14:**
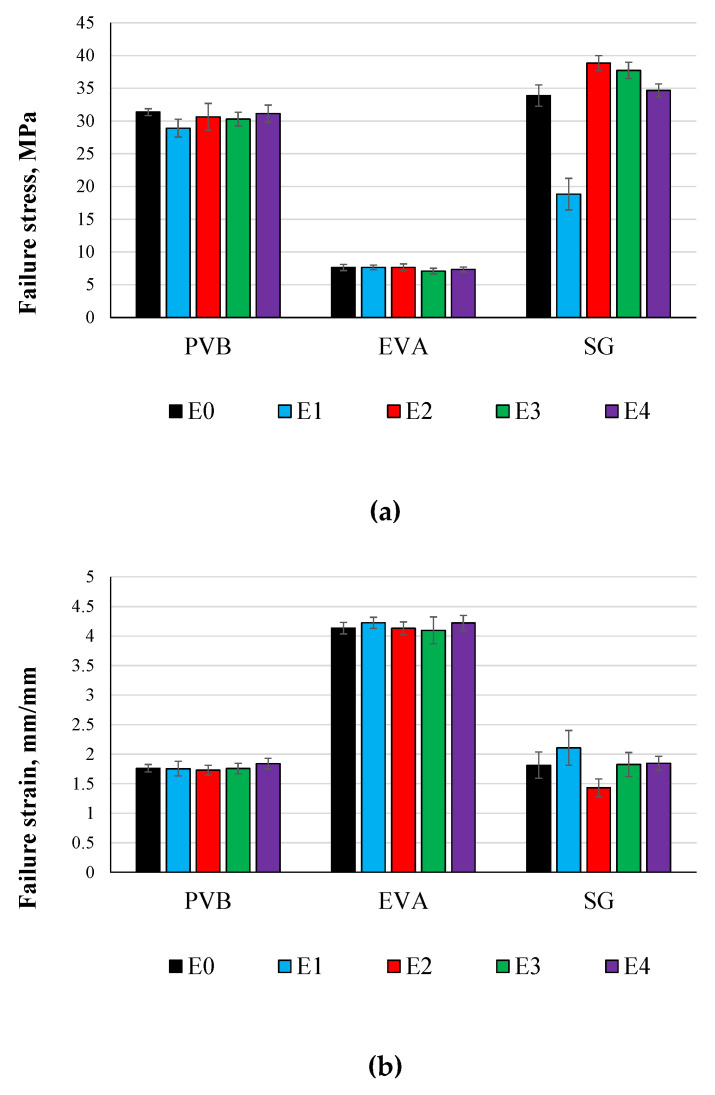

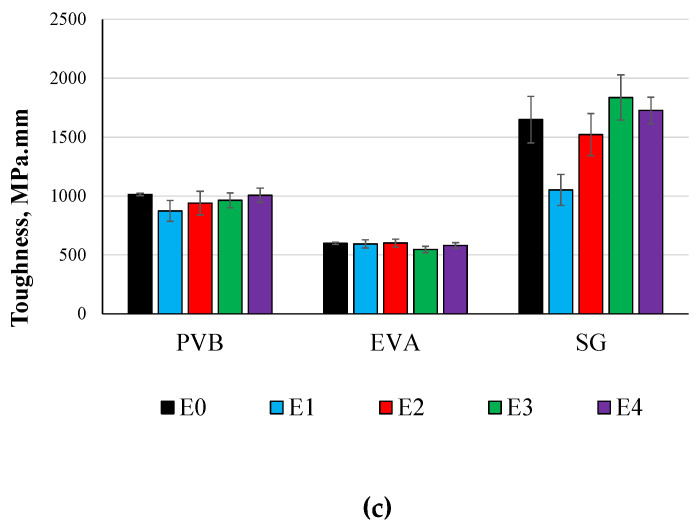
Comparison between Dynamic Testing Results of Accelerated Weathering for Cured Materials: (**a**) Dynamic Testing Failure Stress, (**b**) Dynamic Testing Failure Strain and (**c**) Dynamic Testing Toughness.

Figure 14c shows the maximum toughness of all the materials under the four accelerated weathering environmental effects. In general, PVB is the lowest material in absorbing strain energy and SG5000 is the maximum. Although processed PVB strength is higher than EVA strength, its toughness was lower due to the lower small strain. The most effective environmental effects on the static energy were E4 for the processed PVB, and EVA and E1 for SG5000. It can be concluded that for maximum stress, maximum strain, and toughness, EVA is the most resistive material for the environmental effects. However, PVB was the most affected interlayer material.

Figure 14a shows a comparison between the maximum dynamic failure stress of all the materials under different environmental effects. For the three materials, it can be seen that all the environmental effects caused a loss in strength of processed PVB and EVA, with different percentages. However, some environmental effects cause an increase in the strength of the processed SG5000, unlike the static testing results of processed PVB and EVA, which were not significantly affected by all the studied environmental effects. For processed SG5000, the material strength was significantly affected by E1 and slightly affected by E4, similar to the static behaviors. 

Figure 14b shows the maximum failure strains, where it can be seen that the environmental effects did not cause any significant effect on the failure strain of PVB and EVA. However, for SG5000 the failure strain was decreased significantly by E1. In addition, E2 caused an increase in the failure strain of SG5000.

Figure 14c shows the maximum toughness of all the materials under the four accelerated weathering environmental effects. Unlike the static results, EVA is the lowest material in absorbing strain energy. The most effective environmental effects on the static energy were E4 for PVB, E3 for EVA, and E1 for SG5000. It can be concluded that for maximum stress, maximum strain, and toughness, EVA was the most resistive material.

## 7. Conclusions

In this paper, the environmental effect on the mechanical properties of the virgin and cured/processed polymer interlayer materials was studied. To do that, a survey for the previous research was performed and the literature review was summarized. Based on the survey three polymer interlayer materials were focused, i.e., PVB, EVA, and SG due to their industrial popularity. Testing setups were designed to apply the environmental effects and perform mechanical testing on the polymeric materials.

- The E4 effect most significantly impacts the quasi-static behavior of processed PVB resulting in a decrease in failure stress, failure strain, and toughness;
- The E1, E2, and E3 effects cause softening of the processed PVB and a decrease in failure stress. The responses associated with E1–E3 effects showed lower stress at the same strain values as the E0 control response;
- The environmental effects on the quasi-static response of processed EVA are less significant than the effects on the responses of PVB and SG5000;
- All four environmental effects (E1–E4) caused softening behavior of the EVA resulting in a decrease in failure stresses and negligible impacts on failure strains;
- For SG5000, all four environmental effects (E1–E4) caused a decrease in stiffness in the initial region before pseudo-yielding;
- The E1–E4 effects cause a decrease in failure stress, failure strain, and failure stress relative to the E0 control of the SG5000. The E4 effect impacts the initial region most significantly, while the E1 effect impacts the total response most significantly;
- It can be concluded that ethylene-vinyl acetate represented by EVGARD EVA products is the most resistive product for the environmental effects, while ionomer represented by the Kuraray SG5000 product is the most affected polymer.

## Figures and Tables

**Table 1:** Polymer sheet used in this study.

Material	Manufacturer/Product	Thickness mm (in)	Material State
PVB	Saflex/Standard Clear	0.76(0.03)	Virgin
PVB	Saflex/Standard Clear	1.52 (0.06)	Cured/Processed
EVA	EVGuard	0.76(0.03)	Virgin
EVA	EVGuard	1.52 (0.06)	Cured/Processed
SG5000	Kurary/ SG5000	0.89 (0.035)	Virgin and cured

**Table 2:** Environmental Effects Testing Matrix Stage I.

Effects	Definitions	Duration	Standard
E0	Control Group *	~	~
E1	Humidity- Water Immerse	169 h	ASTM D570 and ISO 62
E2	Constant Temp. 60 °C	16 h	D3045 and ISO 12543
E3	Temp. Cycles- Low Humidity	60 Cycles	
E4	Temp. and Humidity Cycles	20 h	

* For control group E0, no environmental effects were performed. This group is used for comparison to quantify the environmental effects.

**Table 3:** Environmental Effects Testing Matrix Stage II.

Effects	Definitions	Temp, °C	Strain Rate, s^−1^
E0	Control Group *	23	Static, 45
E1	Humidity-Water Immerse		
E2	Constant Temp. 60 °C		
E3	Temp. Cycles-Low Humidity		
E4	Temp. and Humidity Cycles		

* For control group E0, no environmental effects were performed. This group is used for comparison to quantify the environmental effects.

**Table 4:** Specimen label notation.

Sample Name	Material State	Interlayer Type	Test Type
V-P-S	Virgin	PVB	Static
V-P-D	Virgin	PVB	Dynamic
C-P-S	Cured	PVB	Static
C-P-D	Cured	PVB	Dynamic
V-E-S	Virgin	EVA	Static
V-E-D	Virgin	EVA	Dynamic
C-E-S	Cured	EVA	Static
C-E-D	Cured	EVA	Dynamic
V-S-S	Virgin	SG	Static
V-S-D	Virgin	SG	Dynamic
C-S-S	Cured	SG	Static
C-S-D	Cured	SG	Dynamic

## Data Availability

Not applicable.
